# BrHDA6 mediates nonhistone deacetylation of BrSOT12 to positively regulate downy mildew resistance in *Brassica rapa*

**DOI:** 10.1093/hr/uhaf136

**Published:** 2025-05-21

**Authors:** Jianxing Wang, Mei Zheng, Tongbing Su, Bin Zhang, Tao Ma, Xiaojing Liu, Peirong Li, Xiaoyun Xin, Weihong Wang, Xiuyun Zhao, Deshuang Zhang, Yangjun Yu, Jiao Wang, Fenglan Zhang, Wenchao Zhao, Shuancang Yu

**Affiliations:** State Key Laboratory of Vegetable Biobreeding, Beijing Vegetable Research Center (BVRC), Beijing Academy of Agriculture and Forestry Science (BAAFS), Beijing 100097, China; National Engineering Research Center for Vegetables, Beijing Vegetable Research Center, Beijing Academy of Agriculture and Forestry Science, Beijing 100097, China; Beijing Key Laboratory of Crop Molecular Design and Intelligent Breeding, Beijing 100097, China; College of Plant Science and Technology, Beijing University of Agriculture, Beijing 102206, China; State Key Laboratory of Vegetable Biobreeding, Beijing Vegetable Research Center (BVRC), Beijing Academy of Agriculture and Forestry Science (BAAFS), Beijing 100097, China; National Engineering Research Center for Vegetables, Beijing Vegetable Research Center, Beijing Academy of Agriculture and Forestry Science, Beijing 100097, China; Beijing Key Laboratory of Crop Molecular Design and Intelligent Breeding, Beijing 100097, China; State Key Laboratory of Vegetable Biobreeding, Beijing Vegetable Research Center (BVRC), Beijing Academy of Agriculture and Forestry Science (BAAFS), Beijing 100097, China; National Engineering Research Center for Vegetables, Beijing Vegetable Research Center, Beijing Academy of Agriculture and Forestry Science, Beijing 100097, China; Beijing Key Laboratory of Crop Molecular Design and Intelligent Breeding, Beijing 100097, China; State Key Laboratory of Vegetable Biobreeding, Beijing Vegetable Research Center (BVRC), Beijing Academy of Agriculture and Forestry Science (BAAFS), Beijing 100097, China; National Engineering Research Center for Vegetables, Beijing Vegetable Research Center, Beijing Academy of Agriculture and Forestry Science, Beijing 100097, China; Beijing Key Laboratory of Crop Molecular Design and Intelligent Breeding, Beijing 100097, China; State Key Laboratory of Vegetable Biobreeding, Beijing Vegetable Research Center (BVRC), Beijing Academy of Agriculture and Forestry Science (BAAFS), Beijing 100097, China; National Engineering Research Center for Vegetables, Beijing Vegetable Research Center, Beijing Academy of Agriculture and Forestry Science, Beijing 100097, China; Beijing Key Laboratory of Crop Molecular Design and Intelligent Breeding, Beijing 100097, China; State Key Laboratory of Vegetable Biobreeding, Beijing Vegetable Research Center (BVRC), Beijing Academy of Agriculture and Forestry Science (BAAFS), Beijing 100097, China; National Engineering Research Center for Vegetables, Beijing Vegetable Research Center, Beijing Academy of Agriculture and Forestry Science, Beijing 100097, China; Beijing Key Laboratory of Crop Molecular Design and Intelligent Breeding, Beijing 100097, China; State Key Laboratory of Vegetable Biobreeding, Beijing Vegetable Research Center (BVRC), Beijing Academy of Agriculture and Forestry Science (BAAFS), Beijing 100097, China; National Engineering Research Center for Vegetables, Beijing Vegetable Research Center, Beijing Academy of Agriculture and Forestry Science, Beijing 100097, China; Beijing Key Laboratory of Crop Molecular Design and Intelligent Breeding, Beijing 100097, China; State Key Laboratory of Vegetable Biobreeding, Beijing Vegetable Research Center (BVRC), Beijing Academy of Agriculture and Forestry Science (BAAFS), Beijing 100097, China; National Engineering Research Center for Vegetables, Beijing Vegetable Research Center, Beijing Academy of Agriculture and Forestry Science, Beijing 100097, China; Beijing Key Laboratory of Crop Molecular Design and Intelligent Breeding, Beijing 100097, China; State Key Laboratory of Vegetable Biobreeding, Beijing Vegetable Research Center (BVRC), Beijing Academy of Agriculture and Forestry Science (BAAFS), Beijing 100097, China; National Engineering Research Center for Vegetables, Beijing Vegetable Research Center, Beijing Academy of Agriculture and Forestry Science, Beijing 100097, China; Beijing Key Laboratory of Crop Molecular Design and Intelligent Breeding, Beijing 100097, China; State Key Laboratory of Vegetable Biobreeding, Beijing Vegetable Research Center (BVRC), Beijing Academy of Agriculture and Forestry Science (BAAFS), Beijing 100097, China; National Engineering Research Center for Vegetables, Beijing Vegetable Research Center, Beijing Academy of Agriculture and Forestry Science, Beijing 100097, China; Beijing Key Laboratory of Crop Molecular Design and Intelligent Breeding, Beijing 100097, China; State Key Laboratory of Vegetable Biobreeding, Beijing Vegetable Research Center (BVRC), Beijing Academy of Agriculture and Forestry Science (BAAFS), Beijing 100097, China; National Engineering Research Center for Vegetables, Beijing Vegetable Research Center, Beijing Academy of Agriculture and Forestry Science, Beijing 100097, China; Beijing Key Laboratory of Crop Molecular Design and Intelligent Breeding, Beijing 100097, China; State Key Laboratory of Vegetable Biobreeding, Beijing Vegetable Research Center (BVRC), Beijing Academy of Agriculture and Forestry Science (BAAFS), Beijing 100097, China; National Engineering Research Center for Vegetables, Beijing Vegetable Research Center, Beijing Academy of Agriculture and Forestry Science, Beijing 100097, China; Beijing Key Laboratory of Crop Molecular Design and Intelligent Breeding, Beijing 100097, China; State Key Laboratory of Vegetable Biobreeding, Beijing Vegetable Research Center (BVRC), Beijing Academy of Agriculture and Forestry Science (BAAFS), Beijing 100097, China; National Engineering Research Center for Vegetables, Beijing Vegetable Research Center, Beijing Academy of Agriculture and Forestry Science, Beijing 100097, China; Beijing Key Laboratory of Crop Molecular Design and Intelligent Breeding, Beijing 100097, China; State Key Laboratory of Vegetable Biobreeding, Beijing Vegetable Research Center (BVRC), Beijing Academy of Agriculture and Forestry Science (BAAFS), Beijing 100097, China; National Engineering Research Center for Vegetables, Beijing Vegetable Research Center, Beijing Academy of Agriculture and Forestry Science, Beijing 100097, China; Beijing Key Laboratory of Crop Molecular Design and Intelligent Breeding, Beijing 100097, China; College of Plant Science and Technology, Beijing University of Agriculture, Beijing 102206, China; State Key Laboratory of Vegetable Biobreeding, Beijing Vegetable Research Center (BVRC), Beijing Academy of Agriculture and Forestry Science (BAAFS), Beijing 100097, China; National Engineering Research Center for Vegetables, Beijing Vegetable Research Center, Beijing Academy of Agriculture and Forestry Science, Beijing 100097, China; Beijing Key Laboratory of Crop Molecular Design and Intelligent Breeding, Beijing 100097, China

## Abstract

Downy mildew is a major disease that significantly impacts the yield and quality of *Brassica rapa*. While histone deacetylase (*HDAC)* family members are implicated in stress responses, their role in regulating downy mildew resistance in *B. rapa* remains unclear. Herein, we treated the susceptible *B. rapa* line R32 with Trichostatin A (TSA), a potent *HDAC* inhibitor. Notably, TSA application significantly enhanced the susceptibility of *B. rapa* seedlings to downy mildew infection, demonstrating that *HDAC* plays a crucial role in mediating resistance against this pathogen. Subsequently, we conducted phylogenetic analysis of *HDAC* family members and performed high-throughput sequencing to assess *HDAC* gene expression patterns in the resistant (R31) and susceptible (R32) lines following downy mildew inoculation. Notably, the expression of *BrHDA6* was significantly higher in the resistant line R31 compared to the susceptible line R32, suggesting its potential role in disease resistance. Using a genetic transformation system, we generated stable transgenic *B. rapa* plants overexpressing or silenced for *BrHDA6*. Inoculation with the downy mildew pathogen revealed that *BrHDA6* positively regulates disease resistance. Modification omics and parallel reaction monitoring analysis demonstrated that BrHDA6 directly reduces the acetylation level of sulphotransferase 12 (BrSOT12), which likely enhances sulfotransferase activity, consequently boosting salicylic acid production during downy mildew infection. Interaction between BrHDA6 and BrSOT12 was further validated through yeast two-hybrid and dual-luciferase assays. Our study reveals that *BrHDA6* confers downy mildew resistance in *B. rapa* through nonhistone protein deacetylation of BrSOT12, uncovering a novel regulatory mechanism in plant–pathogen interactions.

## Introduction

*Brassica rapa*, as one of the main vegetables grown in China, includes varieties such as Chinese cabbage, pak choi, and turnips, and their cultivation area and yield are increasing each year [1]. Demand for quality products is increasing along with the improvement in people's living standards. It has taken decades for breeders to cultivate high-quality and highly nutritious varieties of *B. rapa*, including Jingqiu No. 3, Jingyan Kuaicai, and Jingchunwa No. 2 [2, 3]. This has resulted in Chinese cabbage, originally a Chinese domestic vegetable, gradually becoming a global vegetable. Unfortunately, pests and diseases limit its field production. Downy mildew is a parasitic oomycete disease mainly occurring on dicotyledonous plants and can be widespread in vegetable crops and field crops. The disease can affect *B. rapa* at any time during its growing period; however, the seedling stage is particularly susceptible [4]. Downy mildew spreads rapid and widely, and in severe cases it can cause 90% of the leaves to rot and dry up. *B. rapa* yield and quality are seriously affected by downy mildew once the leaves become infected.

There has been extensive research related to plant disease resistance and immunity, Yu identified *BrDM*, the primary gene for downy mildew resistance in *B. rapa* seedlings, by fine mapping, and located it in the A8 chromosome. Furthermore, it was revealed that major and minor genes jointly regulate downy mildew resistance in Chinese cabbage [4]. Kim et al.*et al.* detected *BrRHP1* to confer downy mildew resistance on Chinese cabbage [5]. A number of genes, including *BcAF* and *Bcchi*, have been reported to play a role in the resistance to downy mildew of nonheading Chinese cabbage [6]. According to Zhang et al.*et al.*, *BrWAK1* is involved in pattern-triggered immunity responses to downy mildew [7]. Additionally, a long noncoding RNA (lncRNA), *MSTRG.19915* was found directly activated downstream *BrMAPK15*-induced resistance to downy mildew in Chinese cabbage [8].

Phytohormones such as salicylic acid (SA), jasmonic acid (JA), abscisic acid (ABA), and ethylene (ET) play a significant role in plant immune responses [9]. The phytohormone SA is responsible for activating defense responses, primarily against viviparous pathogens, which include the *B. rapa* specific parasites causing downy mildew [10]. In the disease-resistant Chinese cabbage line T12-19, genes associated with the SA pathway were significantly activated upon downy mildew infection. This finding provides the first experimental evidence supporting the role of SA in *B. rapa* resistance against this pathogen [11]. These results align with previous studies by Zhang et al.*et al.*, which demonstrated that downy mildew infection triggers SA accumulation in Chinese cabbage [7, 8]. Collectively, these findings provide compelling evidence that SA serves as a central regulator of defense responses against downy mildew in Brassica crops.

In eukaryotes, histones are important components of chromatin. As part of the process of post-translational modification, methylation, acetylation, phosphorylation, and ubiquitination can alter the structure of chromatin and, in turn, regulate gene expression [11, 12]. Histone acetyltransferases and histone deacetylases (*HDACs*) are responsible for maintaining a dynamic equilibrium of histone acetylation [13]. In plant immunity, *HDACs* are involved in a wide range of biological processes. Furthermore, *HDACs* play an important role in phytohormone signaling pathways, thus facilitating plant growth and maturation [14, 15]. Three *HDACs* families are found in *Arabidopsis*: *RPD3/HDA1*, *Sirtuin*, and *HD2*. The *RPD3/HDA1* family is involved in gene expression, biological processes, and environmental stress responses. Meanwhile, *HDACs* can enhance or suppress disease resistance by directly or indirectly regulating gene expression. Sodium butyrate (NaBT), an inhibitor of *HDACs* that promotes histone acetylation and activates defense-related genes, significantly enhanced rice resistance to rice blast, demonstrating the importance of the *RPD3/HDA1* family in rice immunity [16]. The *HDT701* directly binds to defense-related genes *MAPK6* and *WRKY53* and inhibits their acetylation levels, thereby negatively regulating innate immunity in rice [17]. A subsequent study found that *OsHDA706* plays a role in rice pathogen defense and regulates the jasmonate synthesis pathway [18]. In wheat, TaHDT701 forms a complex with TaHDA6 and TaHOS15 to regulate defense-related genes, ultimately negatively regulating powdery mildew resistance [19]. A plant immune response is also regulated by HDA9, which interacts with HOS15 to regulate the *NLR* gene, an immune receptor within plants [20]. Overexpression of *GhHDA5* in cotton decreased histone acetylation, inhibited the expression of lignin synthesis genes *Gh4CL3* and *GhF5H*, and silencing *GhHDA5* promoted reactive oxygen species (ROS)-related and pathogenesis-related (PR) gene expression. This demonstrated that *GhHDA5* negatively regulates *Verticillium wilt* resistance [21]. A study found that AtERF7 recruits AtHDA19 and AtSin3 complexes to co-repress gene transcription. In *Brassica oleracea*, *AtHDA19* regulates the expression of related genes in response to hormone signaling (JA, ET), while *HDA6* is involved in the induction of JA, ET, and other hormones [22, 23]. Plant basal defense is negatively regulated by *WRKY38* and *WRKY62*, where *WRKY38* and *WRKY62* transcriptions are stimulated by *PstDC3000* and suppressed by *HDA19* [24].

Relevant studies have demonstrated that *HDA6* plays a multifaceted role in plant genome expression, development, and abiotic stress responses [25–27]. Moreover, *HDA6* is an important component of chromatin regulation and stress responses, such as those to drought or salt. Of the three homologs of *HDA6* in yeast RPD3 (*HDA7, 9*, and *19*), *HDA6* has the most homology to *HDA19* [28]. In nonstressed plants, *HDA6* inhibits the direct expression of *SARD1* and *CBP60g* to maintain a low SA level, as well as preventing excessive immune responses that might damage the plant [29]. A novel *HDA6* mutant was reported to be associated with the pathogen response, confirming that *HDA6* is a repressor of pathogen defense in *Arabidopsis* and that it represses and regulates pathogen response genes. This provides theoretical support for the role of *HDA6* in *B. rapa* downy mildew resistance [23].

Considering that downy mildew is the main culprit leading to the reduction of *B. rapa* yield and quality, molecular mechanisms underlying *B. rapa* seedling resistance to downy mildew require investigation. Herein, we investigated downy mildew resistance genes in *B. rapa* seedlings to reveal the genetic mechanisms underlying downy mildew resistance and to develop high-quality resistant varieties.

## Results

### Inhibition of *HDAC* promotes host susceptibility to downy mildew infection

**Figure 1:**
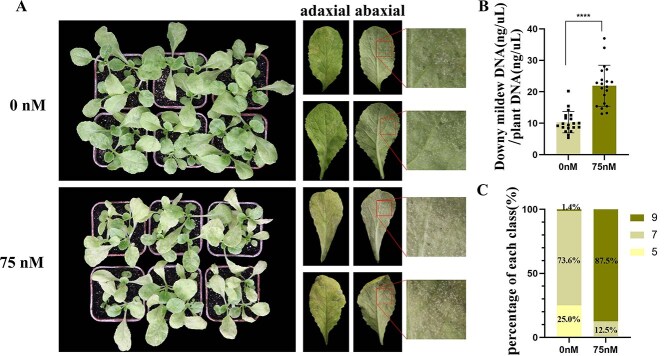
The effect of inhibiting *HDAC* in *B. rapa* on resistance to downy mildew. (A) Phenotypic investigation of downy mildew after 75 and 0 nM TSA treatment. Magnified view (right) of the square region from the left image. (B) The relative content of pathogens in the leaves of the R32 susceptible plant material under 75 and 0 nM TSA treatments of *n* = 24 plants each. Each value represents the mean ± SE of three biological replicates. **** indicates a significant difference between the 75 and 0 nM groups, *P* < 0.0001, as determined using a t-test. (C) Statistics of disease grades under 75 and 0 nM TSA treatments. Each of the experimental and control treatments involved 72 pathogen strains, and the percentage of each grade was calculated.

To investigate whether histone deacetylation plays a role in the response of *B. rapa* to downy mildew, *HDACs* were inhibited by applying histone deacetylation inhibitors before downy mildew inoculation. Trichostatin A (TSA) and NaBT deacetylate *RPD3/HDACs*, resulting in chromatin relaxation, which facilitates the binding of transcription factors and RNA polymerase [30, 31]. Therefore, we selected TSA for the treatment of plant materials R31 and R32. For exploring the optimal concentration of TSA, we set up five gradients in the pre-experiment with concentrations of 0, 25, 50, 75, and 100 nM of TSA sprayed onto the resistance line, followed by inoculation with downy mildew [32]. Upon treatment with 75 nM, the most significant inhibitory effects were observed, as well as the most severe onset of disease (Supplementary Fig. S1). Afterwards, the 75 nM was considered an ideal treatment concentration. In addition, we found that R31 exhibits a robust immune system. Despite TSA treatment leading to pronounced disease symptoms on the leaves, the mold layer on the abaxial side of the leaves was scarcely detectable. As a result, we opted to utilize the disease-susceptible line R32 as the experimental material for this investigation. In this experiment, 24 samples of susceptible *B. rapa* plant material R32 were divided into two groups: one was treated with 75 nM TSA and the other was treated with 0 nM TSA as a control group. Both groups were subsequently inoculated with downy mildew. The results indicated that the concentration of pathogenic fungi in the plants treated with 75 nM TSA was significantly higher than that in the control group (Fig. 1A and B). The assessment was conducted in accordance with the published grading criteria for downy mildew diseases outlined by Yu [4]. Following treatment with 75 nM TSA to inhibit histone deacetylation, 87.5% of the plants were classified as grade 9, while the remaining 12.5% were categorized as grade 7. In contrast, the untreated control group exhibited a disease grade distribution of 5, 7, and 9, with grade 7 being the most prevalent (Fig. 1C). *B. rapa* material R32 was classified as susceptible to downy mildew under control conditions (0 nM). However, after inhibiting *HDAC* gene expression using 75 nM TSA, the disease index increased to 97.22% (Supplementary Fig. S1), placing it in the highly susceptible category. This suggests that deacetylation modifications of endogenous proteins could play a role in modulating the interaction between *B. rapa* and downy mildew.

### Phylogenetic analysis of *HDACs* and expression analysis following inoculation with downy mildew

**Figure 2:**
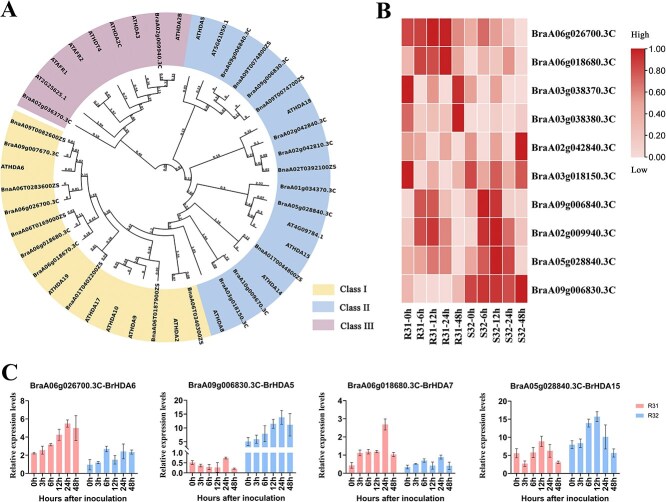
*HDAC* gene expression and evolutionary analysis (A) *B. rapa*, *A. thaliana*, and *B. napus* homologous genes and their evolutionary relationships. (B) Analysis of the differential expression of *HDAC* genes at five time points following inoculation with downy mildew in the resistant (R31) and susceptible (R32) lines using RNA-seq. (C) The expression of *BrHDA6*, *BrHDA5*, *BrHDA7*, and *BrHDA15* in R31 and R32 as analyzed using qRT-PCR using total RNA extracted from leaves at 0, 3, 6, 12, 24, and 48 h postinfection (hpi). Analysis of the qRT-PCR data was performed on samples of the four deacetylase genes at six time points after vaccination with resistant material R31 and susceptible line R32 . The gene encoding actin served as the internal reference. Values represent means ± SD (*n* = 3) from three biological replicates.

To further determine the molecular mechanisms by which *HDACs* regulate the stress response of *B. rapa* to downy mildew, we initially employed the *HDAC* gene sequences from *Arabidopsis thaliana* as queries to identify potential homologous *HDAC* genes within the *B. rapa* genome. This approach yielded a total of 14 candidate *HDAC* genes. After a comprehensive comparison of the classification and evolutionary characteristics of *HDAC* family genes in *B. rapa*, *A. thaliana*, and *Brassica napus* (Canola), we constructed a phylogenetic tree of the *HDAC* family to further identify the specific deacetylase genes involved in downy mildew resistance. *HDACs* are classified into three subfamilies: *RPD3*-like, *HD2*, and *SIR2*-like. Within the *RPD3*-like subfamily (Class I), which is homologous to yeast *RPD3*, members include *HDA2*, *HDA6*, *HDA9*, *HDA10, HDA17*, and *HDA19*. In *B. rapa*, a considerable number of Class I *HDAC* genes are located on chromosome A06, with *BrHDA6* being most closely related to the *HDACs* in *B. napus* found on chromosome 6. Conversely, *ATHDA6* in *A. thaliana* shows greater similarity to its homolog on chromosome 9. Members of Class II include *HDA5*, *HDA8*, *HDA14*, *HDA15*, and *HDA18*. Class III comprises *HD2B*, *HD2C*, and *HDT4*, which are homologous to the yeast sirtuin protein, SIR2 (Fig. 2A).

Subsequently, the expression levels of *HDAC* genes in *B. rapa* were evaluated following inoculation with downy mildew. Resistant line R31 and susceptible line R32 were inoculated with downy mildew, and samples were collected at 0, 3, 6, 12, 24, and 48 h postinoculation for high-throughput RNA sequencing analysis (RNA-seq). Ten deacetylase genes were identified to respond to downy mildew infestation. Through analysis of their expression levels, it was observed that *BrHDA6* exhibited significant differences between susceptible and resistant materials. Specifically, the expression level of *BrHDA6* in the resistant line R31 was notably higher than that in the susceptible line R32 (Fig. 2B). This difference was the most pronounced within the *RPD3/HDACs* family. In contrast, *BrHDA5* showed the opposite result, suggesting its potential as a susceptibility gene. Therefore, *BrHDA6*, which is located on chromosome 6, will be further studied. Quantitative real-time reverse transcription PCR (qRT-PCR) was utilized to validate the *HDAC* genes that responded to downy mildew infection, and the findings were in agreement with the RNA-seq data; i.e. the expression level of *BrHDA6* in the resistant line R31 was significantly higher than that in the susceptible line R32 (Fig. 2C). Recently *HDA5* was reported to function as a negative regulator in *Verticillium wilt* resistance, which further supports the validity of our transcriptome data [21]. Based on the findings from the TSA treatment and subsequent RNA-seq analysis, it was determined that additional in-depth analysis of *BrHDA6* was warranted. We hypothesized that *BrHDA6* might play a role in the immune response to biotic stress. Then, we further analyzed the promoter sequence and coding sequence (CDS) of *BrHDA6* in R31 and R32. The *BrHDA6* CDSs were 100% identical between R31 and R32. Furthermore, protein domain analysis indicated that *BrHDA6* in both genotypes (R31 and R32) possesses characteristic *HDAC* domains, despite their identical genetic background (Supplementary Fig. S2A). To assess whether there is a difference in promoter activity, we conducted dual luciferase (Dual-LUC) assays to analyze the activities of both full-length *BrHDA6* promoters (31-1 and 32-1) and truncated *BrHDA6* promoters (31-2 and 32-2). The results demonstrated that the promoter activities of both full-length and truncated *BrHDA6*-31 were significantly higher than those of their corresponding *BrHDA6*-32 counterparts (Supplementary Fig. S2C). In conclusion, *BrHDA6* plays a positive regulatory role in enhancing the resistance of *B. rapa* to downy mildew.

### *BrHDA6* enhances downy mildew resistance in *B. rapa*

**Figure 3:**
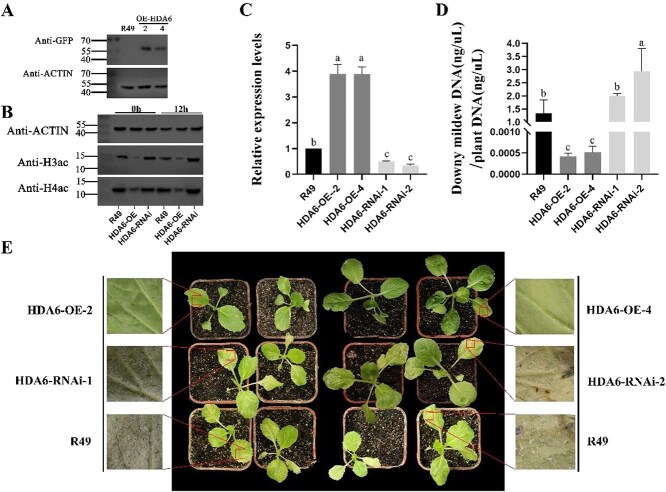
Phenotypes of *BrHDA6* overexpression lines and *BrHDA6*-RNAi lines upon downy mildew infection. (A) Western blotting to detect the positive strain of *BrHDA6*-OE protein carrying the GFP tag. (B) Western blotting to detect the acetylation levels of H3ac and H4ac in R49, *BrHDA6*-RNAi, and *BrHDA6*-OE lines before and after downy mildew inoculation. Actin was applied as an equal loading control. Representative data from three independent experiments. The grayscale value of the color bands was measured using Image J, and the color bands were quantified (Supplementary Fig. S3). (C)The relative mRNA expression in transgenic plants as detected using qRT-PCR. Relative gene expression levels in the R49 line was set to 1.00. Values are the means SEM (*n* = 3). Experiments were repeated three times. (D) Content of downy mildew pathogens in *BrHDA6* overexpression, *BrHDA6*-RNAi, and R49 lines using qRT-PCR. Values represent the means ± SD from three independent experiments. (E) Phenotypes of *BrHDA6* overexpression, RNAi, and R49 lines investigated seven days after inoculation with downy mildew. The images in the squares are magnified.

To determine the specific role of *BrHDA6* in downy mildew resistance, we performed a stable genetic transformation of *BrHDA6*. It is well known that the genetic transformation of *B. rapa* is relatively challenging, largely dependent on the genotype. The inbred line R49, which is susceptible to downy mildew and comparatively easier to carry out the genetic transformation, was selected for this study. Utilizing transgenic technology, we successfully generated *BrHDA6* overexpression and RNA interference (RNAi) plants. Subsequent analysis of RNA and protein levels enabled the identification of overexpressing lines (*BrHDA6*-OE**#**2*, BrHDA6-*OE**#**4) and silenced lines (*BrHDA6*-RNAi**#**1*, BrHDA6*-RNAi**#**2) (Fig. 3A and C). To further elucidate the function of *BrHDA6*, we inoculated both transgenic and wild-type seedlings, which had developed to the two-leaf stage, with downy mildew.

A further investigation was conducted to determine whether *BrHDA6* is capable of histone deacetylation as a result of the stress response to downy mildew pathogen, using antibodies specific for histones H3 (acetyl K4 + K9 + K14 + K18 + K23 + K27) and H4 (acetyl K5 + K8 + K12 + K16). Western blot analysis revealed that *BrHDA6* overexpression reduced histone acetylation levels, while *BrHDA6* silencing elevated acetylation upon downy mildew infection (Fig. 3B). Using ImageJ, we performed a quantitative analysis of the grayscale values of the target bands, revealing that histone acetylation levels in *BrHDA6*-OE plants exhibited a downward trend under steady state or upon downy mildew infection compared to the control group (Supplementary Fig. S3). We propose that *BrHDA6* serve as epigenetic regulators of plant defense against biotic stress, particularly downy mildew infection, by mediating histone acetylation changes.

In terms of phenotypic characteristics, the control, R049, showed distinct downy mildew symptoms, including yellow spots limited by veins on the adaxial side of the leaf and white filamentous mold on the abaxial side, whereas the plants overexpressing *BrHDA6* showed no/low symptoms*.* Similarly, the *BrHDA6*-RNAi plants were found to fall into the susceptible disease class in comparison with the control plants (Fig. 3E). The relative pathogen contents in the *BrHDA6*-OE, *BrHDA6*-RNAi, and wild-type (WT)-R49 plants were subsequently quantified. The results demonstrated that the pathogen levels in the *BrHDA6*-OE plants were significantly lower compared with those in the R49 control plants. Conversely, the pathogen content in the *BrHDA6*-RNAi plants was significantly higher than that in the R49 control plants (Fig. 3D). Genetic and pathological evidence demonstrates that *BrHDA6* functions as a negative regulator of downy mildew infection in *B. rapa*, revealing a novel epigenetic mechanism of disease resistance in *cruciferous* crops.

### Integrated analysis of the proteome and acetylation modification profiles

**Figure 4:**
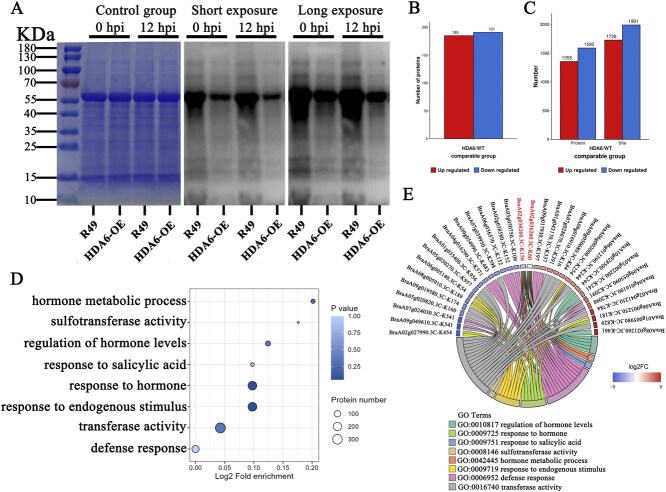
Analysis of Proteomics and Acetylation proteomics. (A) Protein samples from *BrHDA6*-OE, *BrHDA6*-RNAi, and R49 control plants (both pre- and postinoculation with downy mildew) were subjected to Coomassie brilliant blue staining for total protein visualization. Acetylation modification patterns were analyzed using pan-acetyl antibodies under two detection conditions: short exposure mode for baseline acetylation profiling, and long exposure mode to detect subtle changes in acetylation dynamics. (B) Overexpression of differentially expressed proteins (DEPs, Fold change >1.5 and *P*-value <0.05) between *BrHDA6-OE* and WT-R49 in proteome sequencing. The values in the columns indicate the number of proteins that are up or downregulated. (C) The acetylation modification group used a change threshold of significant upregulation as a differential expression amount change exceeding 1.3, and a significant downregulation change threshold of less than 1/1.3, with a *P*-value <0.05 to screen for differentially expressed proteins and differential acetylation modification sites. (D) Pathways associated with the stress response and phytohormones in the acetylation modification enrichment analysis. (E) Proteins and acetylation modification sites involved under stress-responsive and phytohormone-related pathways in the acetylation modification group.

Numerous studies have demonstrated that *HDACs* regulate plant stress responses via deacetylation of both histone and nonhistone proteins. In rice, OsHDA716 coordinates with OsPUB75 to deacetylate OsbZIP46, forming a regulatory module that negatively regulates drought tolerance [33]. We hypothesize that nonhistone acetylation may play a significant role in the defensive response, which has motivated us to pursue further proteomic investigations. To clarify the regulatory effect of downy mildew infection on nonhistone acetylation, we further examined the overall acetylation dynamics of *BrHDA6*-OE and R49 before and after downy mildew stress using western blotting. We first assessed the homogeneity of the samples using Coomassie Brilliant Blue total protein staining to establish a standardized protein loading protocol for *BrHDA6*-OE and R49 transgenic lines. Pan-antibodies were used to detect acetylation levels on global proteins. Comparative analysis revealed significantly reduced global protein acetylation levels in the *BrHDA6* overexpression line relative to the WT-R49 control (Fig. 4A). These findings suggest that *BrHDA6*-mediated nonhistone deacetylation may serve as an important regulatory mechanism contributing to downy mildew resistance in *B. rapa*.

We performed quantitative proteomics and acetylome profiling of *BrHDA6*-OE and R49 plants, and identified 6062 differentially expressed proteins. According to the statistical test *t*-test *P*-value 0.05 as the threshold of significance and by 1.5-fold as the threshold of differential expression change, 376 differentially expressed proteins (DEPs) were identified, of which 185 were upregulated (Fig. 4B). To determine the molecular mechanism by which *BrHDA6* regulates the immune response of *B. rapa* to downy mildew, we conducted acetylation modification analysis based on the proteomics data. Comparative acetylome analysis identified 2945 differentially acetylated proteins, comprising 1355 with significantly increased acetylation levels and 1590 with decreased acetylation. Furthermore, we detected 3718 differential acetylation sites, 1728 of which were upregulated and 1991 of which were downregulated (Fig. 4C). In order to investigate the biological functions of differentially acetylated proteins, we examined gene ontology (GO) term enrichment and found significant enrichment of defense responses and phytohormones, including: (GO:0006950) response to stress; (GO:0006952) defense response; (GO:0009719) response to endogenous stimulus; (GO:0009725) response to hormone; (GO:0009751) response to SA; (GO:0010817) regulation of hormone levels; (GO:0042445) hormone metabolic process; and (GO:0050896) response to stimulus (Fig. 4D). SA plays a crucial role in enhancing resistance of *B. rapa* to downy mildew. Consequently, we investigated the stress response and acetylated proteins within the SA metabolic pathway. A total of 31 differentially abundant proteins (DAPs) and 29 acetylation modification sites were identified (Fig. 4E). These proteins were determined as potential candidates directly regulated by BrHDA6 (Fig. 4E).

**Figure 5:**
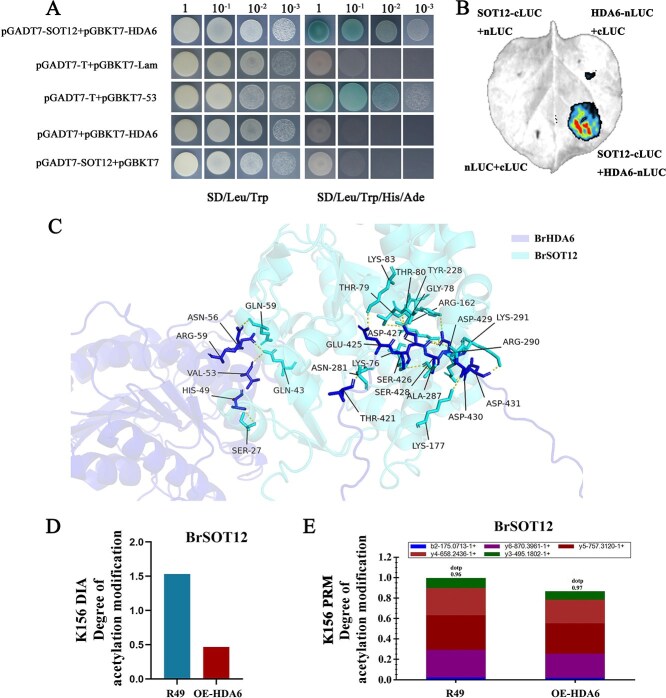
BrHDA6 physically interacts with BrSOT12 to confer resistance against downy mildew. (A) *BrHDA6* and *BrSOT12* were co-expressed in yeast strain AH109, and transformed yeast cells were tested for protein interactions in defective medium at 28°C. Yeast two-hybrid (Y2H) assay showing the BrHDA6–BrSOT12 interaction in yeast. Empty BD and AD were used as the negative controls and pGBKT7-P53 and pGADT7-T were co-transfected as positive controls. (B) BrHDA6-nLuc and BrSOT12-cLuc were co-expressed in *N. benthamiana* leaves, and the interactions between the different combinations were examined using laser confocal microscopy. Dual-Luciferase analysis showing that BrHDA6 and BrSOT12 could interact with each other in *N. benthamiana leaves*. Empty nLuc and cLuc were used as negative controls. (C) Prediction of the interaction pattern of BrHDA6 and BrSOT12 proteins based on the high-throughput intelligent screening technology AlphaFold. (D) Analysis of the degree of acetylation modification of BrSOT12 in *BrHDA6* overexpression and R49 acetylation modification omics. (E) The changes in acetylation modification in *BrHDA6*-OE and R49. Liquid chromatography–tandem mass spectrometry (LC–MS/MS) was used to detect three replicate samples in the parallel reaction analysis (PRM).

### BrHDA6 interacts with and deacetylates BrSOT12

To explore the detailed association of *BrHDA6* with the SA pathway, we validated the screening from section Integrated analysis of the proteome and acetylation modification profiles using a yeast two-hybrid assay. BrHDA6 was used to construct a decoy (bait) vector to validate its interaction with BrSOT12. The results demonstrated that both the positive control and the yeast strains co-transformed with pGBKT7-BrHDA6 and pGADT7-BrSOT12 exhibited growth on QDO/X-a-gal solid medium (Fig. 5A**).** The results demonstrated that BrHDA6 and BrSOT12 are able to interact in yeast cells. Further confirmation of their interaction was required; therefore, we co-expressed the full-length BrHDA6-nLUC and BrSOT12-cLUC in *Nicotiana benthamiana* leaves for a Dual-luciferase assay. Fluorescence was observed under multifunctional fluorescence imaging, and no fluorescence was produced by the three negative controls (Fig. 5B**).** Our results demonstrate a direct interaction between BrHDA6 and BrSOT12, which was further validated through AlphaFold-based structural prediction and molecular docking simulations (Fig. 5C). Modification detection revealed decreased acetylation levels of BrSOT12, suggesting that BrHDA6 plays a role in deacetylating BrSOT12 (Fig. 5D). In the field of proteomics, parallel reaction monitoring (PRM) technology is widely used for quantitative studies [34]. Consequently, we used PRM to quantify the acetylation level of BrSOT12 to further clarify whether BrHDA6 induces BrSOT12 deacetylation. The results indicated that overexpression of *BrHDA6* reduced the acetylation of BrSOT12 to a lower level than that in the control, suggesting a direct regulatory role of BrHDA6 in modulating BrSOT12 acetylation status (Fig. 5E). This evidence suggests that BrHDA6-mediated reduction of BrSOT12 acetylation may modulate plant hormone homeostasis or activate defense-related genes, ultimately enhancing the resistance response of *B.rapa* to downy mildew.

### *BrHDA6* participates in *BrSOT12*-mediated SA synthesis

**Figure 6:**
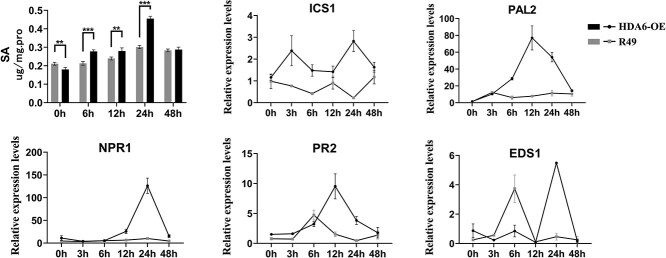
Analysis of the SA content and the expression of SA synthesis-related genes upon downy mildew infection. Time-course profiling of SA accumulation and defense gene expression (*BrICS1, BrPAL2, BrNPR1, BrEDS1, BrPR2*) was performed in inoculated *BrHDA6*-OE and R49 plants across six time points (0–48 hpi). qRT-PCR analysis normalized to *BrActin* expression revealed dynamic transcriptional responses, with all values representing mean ± SD of triplicate biological experiments.

*BrSOT12* belongs to the group of sulfotransferases, and plant sulfation reactions occurring in plants play an important role in processes related to growth and adversity stress [33, 35]. The amino acid sequences of *BrSOT12* and *AtSOT12* were compared, and revealed 85.93% amino acid identity between *B. rapa* and *A. thaliana*, indicating strong evolutionary conservation (Supplementary Fig. S4). The abiotic stress response comprises the involvement of *AtSOT12* in phytohormone signaling pathways, such as those for SA, flavonone, and brassinosteroids [36]. *SOT12* mutations increased SA sensitivity in Chinese cabbage, as identified by genome-wide identification of the sulfotransferase family implicated in abiotic stress [33]. In *A. thaliana*, *AtSOT12* catalyzes the sulfonation of SA, indicating that *AtSOT12* activates SA to regulate pathogen defense responses [37, 38]. We quantified SA levels in transgenic and wild-type plants to assess defense response activation. Time-course analysis (0–48 hpi) demonstrated progressive SA accumulation in both *BrHDA6*-OE and R49 plants following downy mildew inoculation. Notably, *BrHDA6*-OE lines exhibited significantly elevated SA levels compared to R49 controls during the early infection phase (0–24 hpi), suggesting *BrHDA6* enhances early SA-mediated defense signaling (Fig. 6). Concurrently, we analyzed the expression patterns of SA biosynthesis-related genes following pathogen infection. Key defense markers including *NPR1*, *PR2*, and *PAL2* exhibited rapid transcriptional activation within 12 h postinoculation (hpi), displaying distinct upregulation kinetics. It was demonstrated that *BrHDA6* regulates SA signaling-mediated pathogen defense responses by targeting nonhistone protein BrSOT12.

## Discussion

HDACs play a pivotal role in the epigenetic regulation of plant stress responses by mediating the deacetylation of histones and nonhistone proteins. In this study, we successfully identified a *HDAC* gene *BrHDA6* that responds to downy mildew infection and reported its unique functional mechanism in regulating the immune response to downy mildew in *B. rapa*.

### *BrHDA6* enhances resistance of *B. rapa* to downy mildew

*HDA6* plays a pivotal role in mediating plant responses to both abiotic and biotic stresses, with extensive studies documenting its regulatory function in modulating immune pathways under diverse abiotic stress conditions, including salinity, heat, chilling, and drought. [39–41]. Traditionally, *HDA6* has been thought to be an inhibitory factor in the pathogen defense response [23]. In *Arabidopsis*, *HDA6* inhibits and regulates the expression of pathogenesis-related genes *PR1* and *PR2* in *Arabidopsis* and negatively regulates its disease resistance under *Pseudomonas* infection [29]. Nevertheless, HDA6, HDA19, and other proteins form a complex during *Pst DC3000* infection that may inhibit the overexpression of stress-induced genes and reduce the plant's natural defense system [42]. Similar functions have also been found for *HDA9*, a member of the deacetylase family. The HDA9-HOS15 complex prevents H3K9 deacetylation, thereby inhibiting the transcription of *SNC1* and *NLR* genes [43]. These studies reported that *HDA6* participates in immune response as a negative regulatory factor. Our study reveals that histone deacetylation plays a crucial role in *B. rapa's* defense against downy mildew, as evidenced by increased susceptibility in TSA-treated seedlings (Fig. 1A). Expression profiling identified *BrHDA6* as a pathogen-responsive *HDAC*, prompting further investigation of its epigenetic regulatory function (Fig. 2B). Through genetic transformation, we established stable *B. rapa* lines with either *BrHDA6* overexpression or silencing. Pathogenicity assays demonstrated that *BrHDA6* positively regulates downy mildew resistance, with overexpression enhancing and RNAi lines compromising disease tolerance (Fig. 3E). The dynamic regulation of the plant immune response by *HDA6* reveals its central role in disease resistance as well as in growth and development, and breaks with the traditional view that *HDA6* is a negative regulatory factor.

### BrHDA6 modifies the acetylation level of nonhistone BrSOT12 and changes sulfotransferase activity.

Currently, deacetylases are a hot topic in nonhistone targeting research [44]. *HDACs* are involved in the regulation of plant immune responses and the acetylation of nonhistone proteins also affects plant immunity. Generally, acetylation changes on nonhistone proteins are only caused by pathogens that encode acetyltransferases [45]. Indeed, acetylation of nonhistone proteins can modulate protein function through multiple mechanisms, such as promoting protein stability, adjusting enzyme activity, and influencing interactions with other post-translational modifications [44]. Recent studies have revealed that members of the *HDAC* gene family are capable of directly binding to nonhistone proteins to regulate their acetylation levels. High-resolution mass spectrometry combined with acetylation proteomics identified 684 lysine acetylation modification sites in strawberry, revealing the widespread distribution characteristics of many nonhistone acetylation modifications in plants [46]. HDA9 modifies the deacetylation of WRKY53, confirming that *HDA9* is a negative regulator of stress tolerance in plants [47]. The deacetylase FolSir2 from tomato interacts with the nonhistone protein FolGSK3 and specifically catalyzes the deacetylation modification of the FolGSK3 lysine K271 site, significantly increasing the activity of the enzyme and promoting the infection of pathogens [48]. HDA6 shows a similar mechanism for controlling its activity, namely binding to the negative regulatory factor BIN2, deacetylating its K189 site, and altering its activity [49]. HDACs modify specific sites on nonhistone proteins and specifically change the acetylation level of the site, which is valuable for understanding the function of *HDACs* in plant immune responses. Thus, we discovered that transgenic lines displayed significantly reduced global nonhistone acetylation levels compared to wild-type controls under both steady-state and stress conditions. We then integrated the proteome and the modification group, and focused on the analysis of the differentially expressed proteins related to the stress response and the SA metabolic pathway in the modification group. A total of 29 DAPs and 31 acetylation modification sites were identified (Fig. 4E). An intelligent high-throughput screening platform was used to predict candidate protein interactions, and high-scoring differential proteins were selected for yeast two-hybrid verification. The sulfotransferase BrSOT12 was found to interact with BrHDA6 in the yeast two-hybrid and dual-luciferase reporter systems, and a 3D model of the interaction between BrHDA6 and BrSOT12 was predicted (Fig. 5C**).** We then integrated PRM technology and western blotting to confirm that HDA6 reduced SOT12 acetylation under steady-state conditions and that BrHDA6 decreased all protein acetylation relative to R49 under pathogen infection. Our findings suggest that *HDA6*-mediated regulation of nonhistone acetylation forms a crucial component of the epigenetic network governing plant immunity.

### HDA6-SOT12-SA regulates resistance to downy mildew

A wide range of phytohormones is activated in response to stress by plant immune systems, particularly SA, which plays a crucial role in the defense responses of plants. In *Arabidopsis*, SA biosynthesis was mediated through the *AtHDA19* gene, and plants with overexpression of *AtHDA19* (*NPR1*, *SID2*, and *NAHG*) exhibited elevated SA levels and significantly increased expression of SA biosynthesis genes both before and after infection with *Pseudomonas syringae pv. tomato DC3000* [50]*.* It has been reported that downy mildew in *B. rapa* is regulated by SA signaling molecules [7, 51, 52]. There are two metabolic pathways involved in the synthesis of SA, Isochorismate synthase (ICS) and phenylalanine ammonia-lyase (PAL), as well as a regulatory network composed of genes associated with disease resistance, such as *EDS1, PRs, NPR1*, and *PBS3*. Herein, we examined the relative levels of SA in *BrHDA6*-OE and R49 plants at different time points after inoculation with downy mildew. Following infection with the pathogen, SA gradually increased in the *BrHDA6-*OE*, BrPAL1, BrNPR1*, and *BrPR2* plants. Quantification of SA level following downy mildew infection revealed a time-dependent accumulation, with significantly higher SA concentrations in *HDA6*-overexpressing plants compared to R49 during 0–24 hpi. The expression level of *ICS1* gene reached the highest value at 3 and 24 h (Fig. 6). A significant increase in *BrPAL2* gene expression was observed in *B. rapa* during the first 12 h following downy mild infection, it was shown that the ICS and PAL pathways may dominate SA synthesis at different times and places. After synthesis, some SA will exert various biological functions, while the rest will be subjected to enzymatic modification, including glucosylation, methylation, hydroxylation, amination, and sulfonation. Such postsynthesis modification is used to regulate the content of hormones and change their biological activity [53].

Previous studies in *Arabidopsis* have demonstrated that AtSOT12 mediates SA sulfonation, a key post-translational modification that potentiates SA biosynthesis and enhances plant defense responses [37]. Under *Pseudomonas syringae* infestation, overexpression of *ATSOT12* could sulfonate SA, leading to positive feedback regulation of SA production, which protected against pathogenic bacteria invasion [37]. Furthermore, *HDA6* acts as a *HDAC*, which directly regulates SA signaling pathways [23]. Elevated SA levels potentiate the plant's defense response, enabling faster and more robust protection against pathogens. The precise biochemical mechanism remains unresolved whether through direct conversion of SA to 5-sulfosalicylic acid, recruitment of sulfonation cofactors, or suppression of SA degradation pathways. Our current study extends this question to *B. rapa*, where the physiological relevance of SA sulfonation awaits experimental confirmation.

## Conclusion

**Figure 7:**
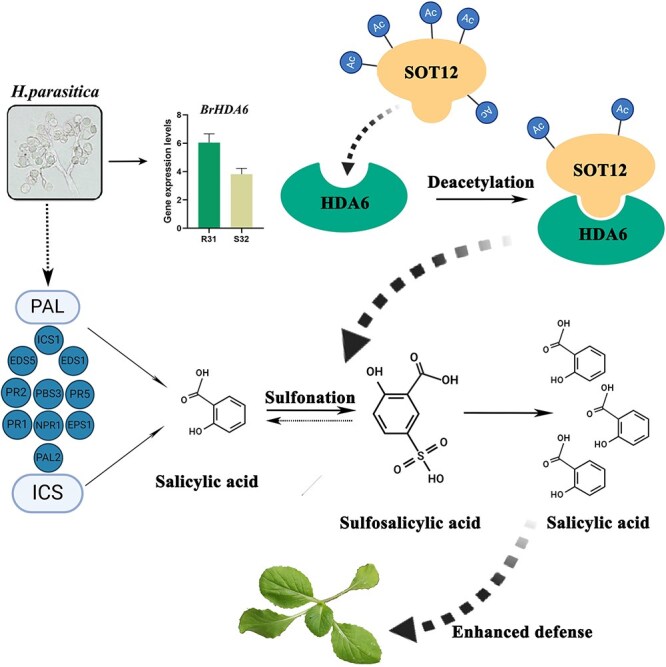
Working model of the BrHDA6-BrSOT12 module in downy mildew resistance of *B. rapa.*

Based on above data, we propose a working model for the BrHDA6-BrSOT12-SA-mediated epigenetic regulatory axis. Our findings demonstrate that BrHDA6 physically interacts with the sulfotransferase BrSOT12 to mediate deacetylation at lysine 156 (K156), a key post-translational modification that activates the SA biosynthetic pathway. This BrHDA6-BrSOT12 regulatory module promotes accelerated SA accumulation, establishing an enhanced defensive state that effectively restricts pathogen colonization (Fig. 7). We speculated that a reduction in BrSOT12 acetylation might alter its enzyme activity or alter protein structure. HDA6 modulates histone and nonhistone acetylation to sustain basal immune surveillance in plants. This regulatory mechanism likely stems from *HDA6* functioning as an epigenetic homeostasis regulator that coordinates energy metabolism for evolutionary adaptation to acute biotic/abiotic perturbations.

## Materials and methods

### Plants materials and growth conditions

*B. rapa* inbred R49, R31, and R32 lines were used in this study. The seeds of *B. rapa* were placed on wet filter paper and germinated at 25°C under full darkness. Once germination was complete, the seeds were sown in the soil, and they were left in a climate chamber with 16 h of light and 8 h of darkness at 23°C. Downy mildew was inoculated when the plant seedlings reached the two-leaf or three-leaf stage (approximately 20 days after sowing).

### Preservation, inoculation, and detection of downy mildew pathogens

*B. rapa* downy mildew is an oomycete parasite that can be propagated at temperatures of 16–24°C and a humidity of 95%. We preserved the downy mildew pathogens in plant susceptible line R32 and changed the host material weekly. The host material R32 was inoculated after 24 h of preincubation under dark and humid conditions.

The humidity is set by a humidifier at a rate of 5 min every 2 h, and the relative humidity of the air is maintained at more than 90% during the humidification process. The pathogens were inoculated on the leaves of the host material R32 by spraying the pathogenic mold in ultrapure water at a concentration of 2 × 10^5^ spores ml^−1^, after which they multiplied and appeared on the leaves abaxially. The inoculated plants were incubated a dark and humid environment for 24 h, followed by a normal incubation (23°C/19°C, day/night), with replacement of host material every other week [52].

Downy mildew pathogens were detected using real-time PCR, according to a method consistent with that published for the identification of Chinese cabbage verticillium wilt pathogens [54]. The DNA of downy mildew pathogens was used as a template and diluted in a 10-fold gradient (1, 10^−1^, 10^−2^, 10^−3^, and 10^−4^) to absolutely quantify the content of pathogens in the plants, and a standard curve was made. The genomic DNA of *B. rapa* was used as a template, diluted twice, and a standard curve was made to absolutely quantify the content of *B. rapa* DNA in samples. The DNA concentration of the sample was normalized using the ACTIN gene as the internal reference, and the relative content of downy mildew pathogens in the samples was calculated as downy mildew pathogen DNA/internal reference gene DNA.

### Resistant (R31) and susceptible (R32) plants were treated with the *HDAC* inhibitor TSA

Plant material was planted in an artificial climate chamber and inoculated with downy mildew fungus once it had grown to the two leaves and one heart stage. TSA at 75 nM was sprayed three times on the adaxial and abaxial sides of the leaves 24 h before inoculation with downy mildew. Subsequent inoculation with downy mildew was consistent with treatment detailed in section Preservation, inoculation, and detection of downy mildew pathogens.

### RNA-sequencing analysis

RNA-sequencing (RNA-Seq) was performed in the Metware Biotechnology Institute (MBI). RNAprep Pure Total RNA Extraction Kit (TIANGEN, Wuhan, China) was used to extract RNA from *B. rapa* leaves. cDNA library was sequenced by Illumina platform. Download the reference genome and its annotation files from the Brassicaceae Database (BRAD, http://www.brassicadb.cn/#/), use HISAT to construct the index, and compare clean reads to the reference genome. Utilize StringTie for novel gene prediction and to assemble complete and accurate transcripts, employ DESeq2 for differential expression analysis between two groups, and apply the Benjamini & Hochberg method for *P*-value adjustment.A Fold Change ≥2 and Adjusted *P*-value ≤0.001 was used to determine the threshold for DEGs. KEGG pathway and GO functional enrichment analysis were implemented by Phyper function in R software.

### Gene cloning (*BrHDA6*-OE and *BrHDA6*-RNAi)

Homologous recombination was used to construct the *BrHDA6* overexpression vector. Download the *BrHDA6* coding region sequence from the Brassicaceae database, use the primers designed by primer5 to amplify the full-length CDS, add the 18 bp overlapping sequence of the homology arm of the P2300-35 s-eGFP vector to both ends of the forward and reverse primers, and deliver it to Sangon Biosynthesis. *BrHDA6* was amplified using plant material R49 cCDNA as a template, and the obtained PCR product was recombinantly ligated with the digested P2300-35 s-eGFP vector through product purification. The recombinant products were transformed into Escherichia coli, and specific primers were used to verify the single clones by colony PCR and sequencing. After sequencing was correct, it was transformed into Agrobacterium tumefaciens to infect R49.

The RNAi interference line of *BrHDA6* was constructed using the pFGC5941 vector. Specific amplification primers were designed based on the CDS region 5 and end 300 bp sequence of *BrHDA6*. Sal I and Xbal restriction sites were inserted into the forward primer, and BamH1 and Kpnl restriction sites were inserted into the reverse primer. The specificity of the sequences was verified online using NCBI Blast and then delivered to Bioengineering for biosynthesis. First, the forward primer is used to amplify the specific interference fragment, and the Sal I and Xbal enzyme digestion vectors are selected. Then the reverse primer is used to amplify the complementary sequence of the interference fragment. The recombinant product of the first step is transformed into E. coli and the correctly sequenced single colony extraction plasmid is selected for the second enzyme digestion system. Then, BamH1 and Kpn1 were used for restriction digestion to form a hairpin structure. After the single colony was sequenced correctly, it was transformed into *Agrobacterium tumefaciens* to infect R49.

### *BrHDA6* promoter activity analysis

We retrieved the sequences of the *BrHDA6* promoter in *B. rapa* from the Brassicaceae Database (BRAD, http://www.brassicadb.cn/#/). Primer Premier 5 (Premier Biosoft, San Francisco, CA, USA) was used to design primer sequences for amplification of the full-length promoter of *BrHDA6* and its fragments. We amplified the *BrHDA6* promotor from R31 and R32 as the 2000 bp upstream of the ATG codon (designated as 31-1 and 32-1, respectively). *BrHDA6* promotor fragments 31-2 and 32-2 were amplified as the 500 bp upstream of the ATG codon. Promotor fragment 31-2 contains an insertion compared with that of 32-2. These promotor fragments were ligated into vector pGreenII0800-LUC. pGreenII0800-LUC-31-1, 32-1, 31-2, and 32-2 were transformed into *Agrobacterium* GV3101 (pSoup). The four bacterial solutions were diluted with resuspension buffer (10 mM MES,10 mM MgCl_2,_ 200 μM AS and ddH_2_O), and the concentration of P19 was adjusted to OD_600_ = 1, and the four *Agrobacterium* suspensions were added to P19 and mixed evenly and injected into *N. benthamiana* leaves. Following incubation in the dark for 24 h and under normal light conditions for 24 h, the leaves were smeared with Beetle Luciferin and incubated in the dark for 5 min. Fluorescence intensity was measured using a multifunctional imaging system (Fusion-FX7, Vilber, Marne-la-Vallée cedex 3, France).

### Quantitative real-time reverse transcription PCR

For qRT-PCR, total RNA was isolated using an RNeasy Plant Mini Kit (TIANGEN, Beijing, China). RNA was reverse transcribed to cDNA using HiScript III RT SuperMix for qPCR (Vazyme, Nanjing, China). The real-time PCR step was performed using a Cham Q Universal SYBR qPCR Master Mix (Vazyme) on a Roche Light Cycler® 480 II instrument (Roche, Basel, Switzerland). Amplification was performed using the following program: Pre-denaturation at 95°C for 5 min; followed by 45 cycles of denaturation at 95°C for 10 s, primer annealing at 60°C (the annealing temperature varied depending on the gene-specific primers) for 10 s, and then primer extension at 72°C for 10 s. The relative expression of the target genes was calculated using the 2^−∆∆Ct^ method [55].

### Proteomics and acetylation proteomics enrichment analysis

Proteomics, acetylated proteomics, and PRM were performed by PTM Bio (Chicago, IL, USA), and plant material for *BrHDA6*-OE, *BrHDA6*-RNAi, and R49 were the same sample. Under the same treatment, the abundance of a certain protein did not change, while its modification level changed significantly. The changes in the degree of acetylation modification of a protein under the current treatment affected the protein’s activity without being influenced by changes in protein abundance in response to stress.

### PRM

The PRM technology achieves targeted relative or absolute quantification of target proteins/modified peptides by selectively detecting specific peptide segments or target peptide segments. Firstly, the selection capability of a quadrupole mass analyzer (such as the Orbitrap series) was utilized to select the precursor ions of the target peptide segments in Q1. Subsequently, the precursor ions were fragmented in the collision cell. Finally, a high-resolution, mass-accurate analyzer was used to detect information about all the fragments within the selected precursor ion windows in the secondary mass spectrometry. In this study, a specific analysis of the acetylation modification level of the target protein BrSOT12 in *BrHDA6*-OE and WT R49 lines was conducted.

### Dual-luciferase assay and Y2H assays

The GV3101 strain was generated by transformation with PCAMBIA1300-nLUC and PCAMBIA1300-cLUC vectors ligated with *BrHDA6* and *BrSOT12* full-length CDS sequences, respectively. Positive clones were stored at 28°C for 16 h, collected, resuspended in infestation solution, and their OD_600_ values were adjusted to be the same. After dark and normal incubation (23°C/19°C, day/night) for 24 h, a luminescent substrate was applied to the surrounding part of the injected infection solution of leaves for fluorescence detection.

*BrHDA6* and *BrSOT12* CDS sequences were inserted into pGBKT7 and pGADT7 vectors for bait and prey vectors, respectively. The resulting vectors were co-transformed into the yeast strain AH109. SD/-Leu-Trp and SD/-Leu-Trp-His-Ade deficient medium were used for selection. Positive clones co-transformed with BrHDA6 and BrSOT12 were able to grow in the four-amino acid deficient medium after three selection experiments, with similar results.

### Protein extraction and western blotting

Leaves of *BrHDA6*-OE, *BrHDA6*-RNAi, and R49 plants were powdered. Antibodies recognizing histone H3 and H4, and pan-antibodies were used to detect acetylation, with anti-GFP antibodies as controls. Powdered samples from three positive lines of *BrHDA6*-OE and *BrHDA6*-RNAi were mixed. Proteins were extracted using Lysis Buffer for WB Assays (Yeasen, Shanghai, China) and Protease Inhibitor Cocktail (Beyotime, Shanghai, China), heated for 15 min in boiling water, immediately placed on ice for 5 min, and centrifuged at 4°C for 10 min. Proteins in the supernatant were separated using a polyacrylamide gel, followed by transfer to a PVDF membrane. The membrane was incubated with primary and secondary antibodies and washed with 1 × TBST buffer. The pan-antibodies were diluted at 1:1000, while the other antibodies were diluted at 1:10 000. The immunoreactive proteins on the membrane were visualized using an Enhanced Chemiluminescent Substrate Kit (Yeasen, Shanghai, China).

### SA treatments

Overexpressed *BrHDA6* and WT-R49 grown to about 20 days old were inoculated with downy mildew, and the inoculation method was referred to 4.2. The *B.rapa* leaves of *BrHDA6*-OE and WT-R49 were sampled at 0, 3, 6, 12, 24, and 48 h after inoculation and quickly frozen in liquid nitrogen. Three leaves were pooled in each sample. The samples used were detected using a plant SA ELISA kit (Saipei, Wuhan, China), and the specific method was referred to the kit instructions.

## Supplementary Material

Web_Material_uhaf136

## Data Availability

Material and data availability All data required for evaluating the conclusions in the paper are available. All materials generated in this study are available from the corresponding author S.C.Y.
