# Supplementary material for: BrHDA6 mediates nonhistone deacetylation of BrSOT12 to positively regulate downy mildew resistance in Brassica rapa
Source: Hortic Res. 2025 May 21;12(8):uhaf136. doi: 10.1093/hr/uhaf136 (PMC12272844; doi:10.1093/hr/uhaf136)
Supplement: Web_Material_uhaf136 [file web_material_uhaf136.zip › Supplementary information.docx]

**Supplementary Fig. S1**

(A) Phenotypic investigation of downy mildew inoculation after treating R31 with different concentrations of TSA (B) Relative content of downy mildew pathogens in R31 (C) Disease index under 75 nM and 0 nM TSA treatments based on disease levels. Each experiment tested at least 24 plants. Data show the means ± SE of three biological replicates.

**Supplementary Fig. S2** Conserved domains and promoter activity of *BrHDA6* in resistant and susceptible materials

(A) The *BrHDA6* CDS region was amplified in the R31 and R32 lines, and the conserved domain differences were predicted at the NCBI ([National Center for Biotechnology Information](https://www.ncbi.nlm.nih.gov/)) (B) Alignment of the 2000 bp *BrHDA6* promoter sequence from resistant and susceptible materials. Pro31-1 and Pro32-1 are the full length promoter sequences. Pro31-2 and Pro32-2 represent the 500 bp upstream of the *BrHDA6* start codon. (C) Pro31-1, Pro32-1, Pro31-2, Pro32-2 fluorescence intensity. Fluorescence intensity was measured using a multifunctional imaging system (Vilber Fusion-FX7). (D) Absolute quantification of fluorescence values in Pro31-1, Pro32-1, Pro31-2, and Pro32-2 was performed using a multifunctional imaging system. Data show the means ± SE of three biological replicates.

**Supplementary Fig. S3** Western blotting to detect the acetylation levels of H3ac and H4ac in R49, *BrHDA6*-RNAi, and *BrHDA6*-OE lines before and after downy mildew inoculation. Actin was applied as an equal loading control. Representative data from three independent experiments. The grayscale value of the color bands was measured using Image J, and the color bands were quantified.

**Supplementary Fig. S4** AtSOT12 amino acid sequence similarity with BrSOT12

The amino acid sequences of AtSOT12 were obtained from the Arabidopsis database ([TAIR - Home](https://www.arabidopsis.org/)), and the amino acid sequences of BrSOT12 were obtained from the brassicaceae database (BRAD, http://www.brassicadb.cn/#/). DNAMAN Software was used for sequence alignment.
