# Supplementary figures and images for: BrHDA6 mediates nonhistone deacetylation of BrSOT12 to positively regulate downy mildew resistance in Brassica rapa
Source: Hortic Res. 2025 May 21;12(8):uhaf136. doi: 10.1093/hr/uhaf136 (PMC12272844; doi:10.1093/hr/uhaf136)

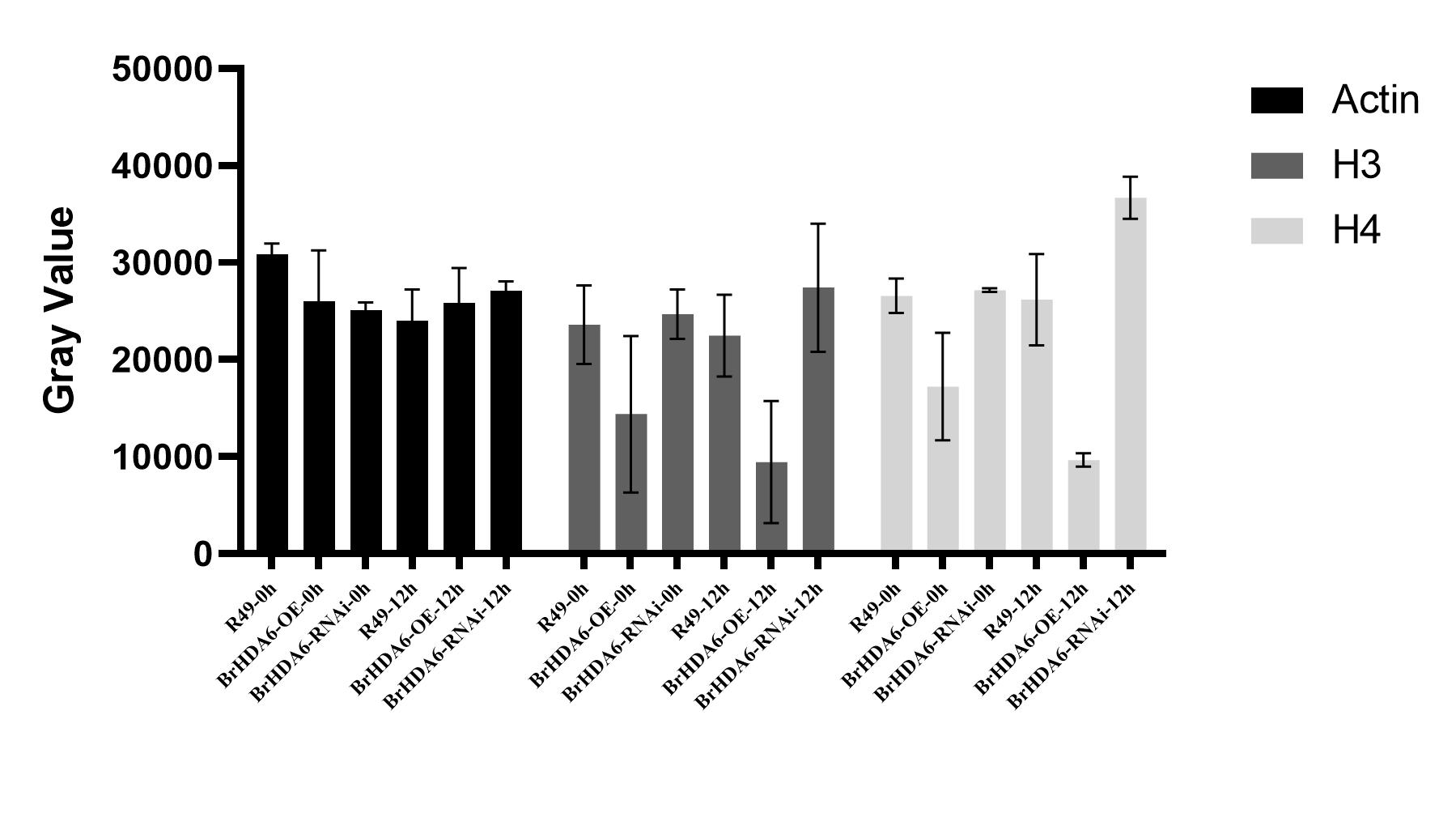

Supplement: Web_Material_uhaf136 [file web_material_uhaf136.zip › Supplementary Figure S3.jpg]

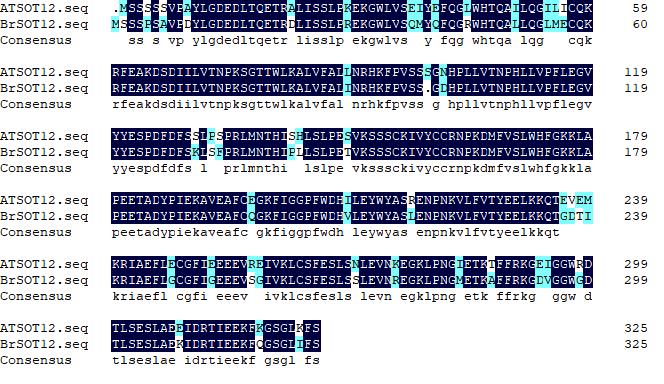

Supplement: Web_Material_uhaf136 [file web_material_uhaf136.zip › Supplementary Figure S4.jpg]
